# Hepatitis A Exposure Response and Outbreak Prevention in a Large Urban Jail — Los Angeles County, California, May–July 2023

**DOI:** 10.15585/mmwr.mm7306a3

**Published:** 2024-02-15

**Authors:** Nazia S. Qureshi, Alma J. Villatoro, Ngoc Dung T. Tran, Sulma J. Herrera, Stephen P. Judge, Ling Fang, Sean O. Henderson, Kristy A. Stanley

**Affiliations:** ^1^Integrated Correctional Health Services-Los Angeles County Department of Health Services, Los Angeles, California; ^2^Division of Infectious Diseases, Harbor-UCLA Medical Center, Torrance, California.

**Summary**

**What is already known about this topic?**

Risk for hepatitis A transmission in correctional settings is high because of the high proportion of homelessness and injection drug use among persons who are incarcerated.

**What is added by this report?**

On May 30, 2023, the Los Angeles County Jail system was notified that an incarcerated person had received a positive hepatitis A test result. Using electronic health records and the state immunization registry, investigators identified persons eligible for hepatitis A vaccination, and a vaccination response was initiated within 48 hours: 2,766 persons were offered vaccine, and 1,510 (54.6%) agreed to receive it. No additional cases were identified.

**What are the implications for public health practice?**

Identifying contacts promptly and using immunization and serology records to ensure rapid delivery of postexposure prophylactic vaccine can help prevent hepatitis A transmission during exposures among incarcerated populations.

## Abstract

Correctional settings provide a high-risk environment for hepatitis A transmission because of the high proportion of homelessness and injection drug use among persons who are incarcerated. On May 30, 2023, Los Angeles County Department of Public Health informed the Communicable Disease Surveillance and Control (CDSC) unit of the Los Angeles County Jail system that a symptomatic incarcerated person had received a positive test result for acute hepatitis A. Upon learning the next day that the patient was a food handler, CDSC staff members identified 5,830 potential contacts of the index patient, 1,702 of whom had been released from the jail. During June 1–12, a total of 2,766 contacts who did not have a documented history of hepatitis A serology or vaccination that could be confirmed from the electronic health record or state immunization registry were identified. These persons were offered hepatitis A vaccination as postexposure prophylaxis; 1,510 (54.6%) accepted vaccination. Contacts who were food handlers without confirmed evidence of immunity and who declined vaccination were removed from food-handling duties for the duration of their potential incubation period. No additional cases were identified. Identifying contacts promptly and using immunization and serology records to ensure rapid delivery of postexposure prophylactic vaccine can help prevent hepatitis A transmission during exposures among incarcerated populations.

## Investigation and Results

The Los Angeles County Jail system (LACJ), the largest in the United States, consists of six facilities. The average number of bookings per year is 53,000, and daily census is approximately 13,330 persons. Correctional Health Services (CHS), a department within the Los Angeles County Department of Health Services, provides health care for the incarcerated population.

### Index Patient

**FIGURE 1:**
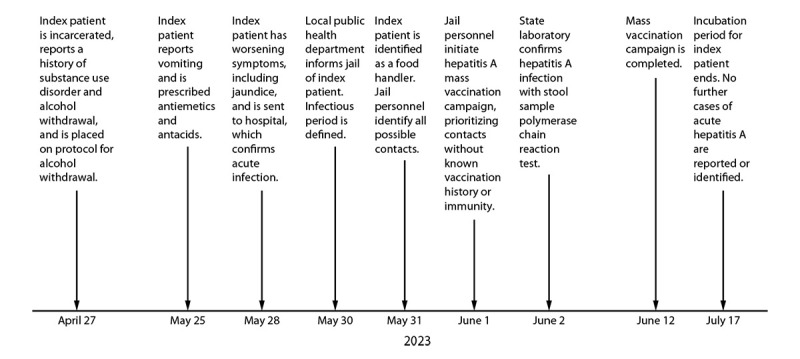
Timeline of hepatitis A exposure discovery and response by Correctional Health Services Communicable Disease and Surveillance Unit staff members — Los Angeles County, California, April–July 2023

On May 25, 2023, an incarcerated man aged 41 years housed in the Los Angeles County Men’s Central Jail sought care at a clinic and reported vomiting for 2 days (Figure 1). The clinic documented that he received antiemetics and antacids and that he reported feeling better later that day. On May 28, he sought care at LACJ Urgent Care, reporting that he had not eaten in 4 days because of abdominal pain, nausea, and vomiting, and that he had jaundice. LACJ Urgent Care staff members noted jaundice of the skin and that the patient had been incarcerated on April 27, 2023, with self-reported homelessness, injection drug use, and alcohol use disorder on the intake history. He had been on a Clinical Institute Withdrawal Assessment protocol beginning April 28, 2023, and was transferred to Los Angeles General Medical Center (LAGMC) from LACJ Urgent Care on May 28 for emergency evaluation. He remained there until June 2. Liver enzymes were elevated, and antihepatitis A virus (HAV) immunoglobulin (Ig) M was reactive. A stool sample collected on May 28 was positive for hepatitis A by polymerase chain reaction on June 2. The patient had no documented history of hepatitis A immunity (vaccination or serology) in the existing electronic health records or in the statewide immunization registry.*

### Exposure Determination

On May 30, CHS Communicable Disease Surveillance and Control staff members were informed of the reactive anti-HAV IgM test result and formulated a plan to provide postexposure prophylactic hepatitis A vaccination to persons who had shared housing with the index patient during the infectious period.^†^ Based on the reported symptoms of the index patient, the index patient’s infectious period was defined as May 9–28, with the potential incubation period of the index patient’s contacts estimated to end on July 17.^§^ On May 31, the Acute Communicable Disease Control (ACDC) branch of the Los Angeles County Department of Public Health informed CHS that they had interviewed the patient earlier the same day during his inpatient stay at LAGMC, and he had been assigned to food preparation in the Men’s Central Jail kitchen. The contact investigation was expanded to account for both shared housing and food handling after the interview with the index patient.

## Public Health Response

**FIGURE 2:**
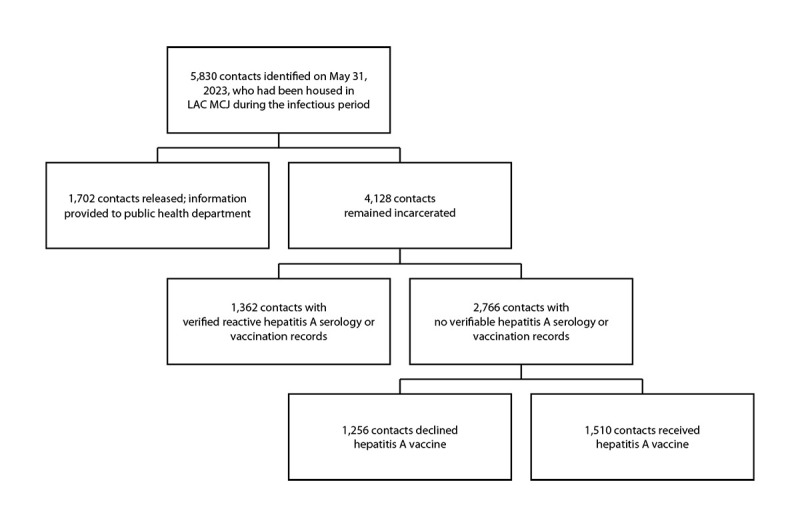
Identification of contacts at Los Angeles County Men’s Central Jail who were eligible to receive postexposure prophylaxis hepatitis A vaccine — Los Angeles County, California, May–July 2023 **Abbreviations:** LAC = Los Angeles County; MCJ = Los Angeles County Men’s Central Jail.

CHS Communicable Disease Surveillance and Control staff members identified and shared with ACDC a list of 5,830 persons who had been housed in Men’s Central Jail during the defined infectious period, 1,702 of whom had been released from the jail. From the list of 4,128 contacts in custody, electronic health records and the state immunization registry were reviewed to remove persons with documented positive hepatitis A serology or vaccination. (Figure 2). This activity was reviewed and approved by the Los Angeles County Public Health, Ambulatory Care Network, and Health Services Administration Institutional Review Board.^¶^

### Vaccination

An initial hepatitis A vaccine supply was procured from the Los Angeles County Department of Public Health (226 doses), and CHS purchased additional vaccine (1,500 doses). Because of the initial limited supply, CHS began offering vaccine on June 1 only to persons located in the same housing units as the index patient, and then, on June 2 and June 3, to those in the additional Men’s Central Jail kitchen incarcerated worker dormitories. Upon acquisition of additional vaccine, vaccination of the remaining contacts (i.e., persons who were incarcerated who had been in Men’s Central Jail during the infectious period and who were not kitchen workers) began on June 4. During June 1–12, a total of 2,766 persons were offered vaccine and 1,510 (54.6%) agreed to receive it. Persons who initially declined vaccination were offered a second opportunity to receive vaccine. Incarcerated kitchen workers with undocumented vaccination history or undocumented serology who declined vaccination were removed from kitchen duties until the end of their potential incubation period.

Los Angeles County Men’s Central Jail health care and custodial employees who were in contact with the index patient during his infectious period were notified of possible exposure and were offered hepatitis A vaccination through a combination of CHS employee health clinic and Los Angeles County Department of Public Health immunization services. Daily CHS communicable disease surveillance laboratory reports that identified reactive anti-HAV IgM results were enhanced by creating an additional report that noted hepatitis A or any related signs or symptoms as a reason for emergency hospital transfer. As of October 16, 2023, no additional cases of acute hepatitis A had been reported or identified in any of the LACJ facilities.

## Discussion

Since 2016, person-to-person outbreaks of hepatitis A in the United States have been increasingly occurring among persons who use drugs, those who experience homelessness, and men who have sex with men (*1*). The risk for hepatitis A transmission is elevated in jails because they house a disproportionate number of persons in these populations, in addition to their crowded living conditions and transient population. Identification of an acute hepatitis A case in a jail, therefore, requires a prompt response and contact identification (*2*).

CHS was able to implement a timely infection control response by identifying all possible incarcerated contacts and initiating a mass vaccination response within 48 hours of notification of the index case (Figure 1). Mass vaccination campaigns for time-sensitive responses in jail settings can be challenging because they involve a large number of persons, as well as logistic issues, and obstacles to timely vaccine procurement. During 2007–2010, hepatitis vaccination campaigns were conducted at LACJ among men who have sex with men (*3*) and during 2017–2019 among the entire LACJ population (CHS, unpublished data, 2019) in response to the 2017 hepatitis A outbreak in San Diego (*4*). 

An effort to improve compliance with the mandatory reporting to the California immunization registry led to steps being taken to improve the quality and completeness of hepatitis A and B vaccination records in the state immunization registry as well as hepatitis A and B vaccination and serology records in the electronic health record. These records helped focus vaccination efforts on persons who were not immune and offer postexposure prophylaxis to those identified as eligible for receipt within 2 weeks of identifying the index patient. The prompt vaccine rollout likely helped reduce transmission and prevent an outbreak among the LACJ population, and the enhanced surveillance, which included the monitoring of emergency hospital transfers made because of suspicion of acute hepatitis A, helped identify possible secondary cases or clusters needing further investigation. Because of the range of the hepatitis A incubation period (15–50 days) and the date of incarceration of the index patient, whether his infection was acquired before or during incarceration is uncertain. The index patient had reported risk factors at the time of intake (i.e., homelessness and injection drug use) for which hepatitis A vaccination is recommended (*1*,*2*). The correctional environment presents a unique opportunity to reduce hepatitis A transmission and disease through vaccination (*5*–*7*); accordingly, CHS might consider a more comprehensive routine vaccination strategy, including offering vaccination at intake.

### Implications for Public Health Practice 

The infection control response initially included a plan to offer hepatitis A vaccine and Ig to persons who were immunocompromised, aged >60 years, or both (*8*); however, Ig could not be obtained within the indicated time frame because of logistic issues. Future infection control planning at CHS involves maintaining a supply of hepatitis A Ig for emergency use in case of an exposure or outbreak. A major limitation of the CHS hepatitis A surveillance process was that reactive IgM anti-HAV laboratory results from LAGMC did not appear in CHS communicable disease laboratory reports because of the different electronic health record identifiers used by the two facilities. A modified communicable disease surveillance report that retrieves reactive IgM anti-HAV results from LAGMC conducted on CHS patients that contains CHS-specific identifiers was created after the response to help prevent delays in identifying cases and planning for exposure response and mitigation. This exposure response highlights the importance of initiating a rapid response to hepatitis A exposure in a jail setting to minimize risk for transmission and help prevent an outbreak. Having relevant laboratory results for reportable communicable diseases consistently and seamlessly communicated electronically across different health systems with mutual patients and using serology and vaccination records from electronic health records and state immunization registries can facilitate and optimize the response to a potential exposure by ensuring the timely administration of postexposure prophylaxis to those who are at greatest risk.

## Footnotes

* Since 2000, all persons incarcerated within LACJ have electronic health records. As of September 2022, CHS began using the same electronic health records system as the Los Angeles County Department of Health Services.

† Although CHS and LAGMC share the same electronic health records platform, the electronic health record identifiers used by the two facilities are different and led to reliance on external notification of the reactive IgM anti-HAV test result.

§
https://www.bop.gov/resources/pdfs/hep_a_timeline_calc.xlsx

¶ 45 C.F.R. part 46.101(c); 21 C.F.R. part 56.

## References

[1] CDC. Outbreaks of hepatitis A across the United States. Atlanta, GA: US Department of Health and Human Services, CDC; 2023. https://www.cdc.gov/hepatitis/outbreaks/2017March-HepatitisA.htm

[2] Federal Bureau of Prisons. Hepatitis A: clinical guidance. Washington, DC: United States Department of Justice, Federal Bureau of Prisons; 2019. https://www.bop.gov/resources/pdfs/hepatitis_a_cpg_112019.pdf

[3] Costumbrado J, Stirland A, Cox G, Implementation of a hepatitis A/B vaccination program using an accelerated schedule among high-risk inmates, Los Angeles County Jail, 2007–2010. Vaccine 2012;30:6878–82. 10.1016/j.vaccine.2012.09.006 22989688

[4] San Diego County Health & Human Services Agency. 2017 hepatitis A outbreak. San Diego, CA: San Diego County Health & Human Services Agency; 2017. https://www.sandiegocounty.gov/content/sdc/hhsa/programs/phs/community_epidemiology/dc/Hepatitis_A/outbreak.html

[5] Gondles EF. A call to immunize the correctional population for hepatitis A and B. Am J Med 2005;118(Suppl 10A):84–9. 10.1016/j.amjmed.2005.07.025 16271547

[6] Vong S, Fiore AE, Haight DO, Vaccination in the county jail as a strategy to reach high risk adults during a community-based hepatitis A outbreak among methamphetamine drug users. Vaccine 2005;23:1021–8. 10.1016/j.vaccine.2004.07.038 15620475

[7] Hagan LM, Montgomery MP, Lauro PL, Recent incarceration among individuals infected with hepatitis A virus during person-to-person community outbreaks, United States, 2016–2020. Public Health Rep 2023;138:619–24. 10.1177/00333549221108413 35856418 PMC10291163

[8] Gounder P, Balter S. Rx for prevention: clinical recognition and management of hepatitis A: preventing outbreaks in Los Angeles County. Los Angeles, CA: County of Los Angeles Public Health; 2019. http://rx.ph.lacounty.gov/RxHepA1119

